# Cell-Specific “Competition for Calories” Drives Asymmetric Nutrient-Energy Partitioning, Obesity, and Metabolic Diseases in Human and Non-human Animals

**DOI:** 10.3389/fphys.2018.01053

**Published:** 2018-08-10

**Authors:** Edward Archer, Gregory Pavela, Samantha McDonald, Carl J. Lavie, James O. Hill

**Affiliations:** ^1^Evolving FX, Jupiter, FL, United States; ^2^The University of Alabama at Birmingham, Birmingham, AL, United States; ^3^East Carolina University, Greenville, NC, United States; ^4^School of Medicine, John Ochsner Heart and Vascular Institute, The University of Queensland, New Orleans, LA, United States; ^5^Center for Human Nutrition, University of Colorado Health Sciences Center, Denver, CO, United States

**Keywords:** obesity, nutrition, physiology, non-genetic, evolution, competition

## Abstract

The mammalian body is a complex physiologic “ecosystem” in which cells compete for calories (i.e., nutrient-energy). Axiomatically, cell-types with competitive advantages acquire a greater number of consumed calories, and when possible, increase in size and/or number. Thus, it is logical and parsimonious to posit that obesity is the competitive advantages of fat-cells (adipocytes) driving a disproportionate acquisition and storage of nutrient-energy. Accordingly, we introduce two conceptual frameworks. *Asymmetric Nutrient-Energy Partitioning* describes the context-dependent, cell-specific *competition for calories* that determines the partitioning of nutrient-energy to oxidation, anabolism, and/or storage; and *Effective Caloric Intake* which describes the number of calories available to constrain energy-intake via the inhibition of the sensorimotor appetitive cells in the liver and brain that govern ingestive behaviors. Inherent in these frameworks is the independence and dissociation of the energetic demands of metabolism and the neuro-muscular pathways that initiate ingestive behaviors and energy intake. As we demonstrate, if the sensorimotor cells suffer relative caloric deprivation via asymmetric competition from other cell-types (e.g., skeletal muscle- or fat-cells), energy-intake is increased to compensate for both *real* and merely *apparent* deficits in energy-homeostasis (i.e., true and false signals, respectively). Thus, we posit that the chronic positive energy balance (i.e., over-nutrition) that leads to obesity and metabolic diseases is engendered by *apparent* deficits (i.e., *false signals)* driven by the asymmetric inter-cellular *competition for calories* and concomitant differential partitioning of nutrient-energy to storage. These frameworks, in concert with our previous theoretic work, the *Maternal Resources Hypothesis*, provide a parsimonious and rigorous explanation for the rapid rise in the global prevalence of increased body and fat mass, and associated metabolic dysfunctions in humans and other mammals inclusive of companion, domesticated, laboratory, and feral animals.

## Introduction

“Frustra fit per plura, quod potest fieri per pauciora” [It is futile to do with more that which can be done with less]. William of Occam (Thorburn, 1918)

Although obesity is described as a complex phenomenon of disputed etiology (Archer et al., 2018), the defining characteristic is an excess of body-fat mass (Schwartz et al., 2017) attributable to a greater number and/or size of fat-cells (adipocytes) relative to other cell-types (Brook et al., 1972; Salans et al., 1973; Knittle et al., 1979; Sjostrom and William-Olsson, 1981). Thus, it is logical and parsimonious to posit that the etiology of obesity is simply the result of physiologic processes that increase fat-cell number, size, or both. Since it is well-established that *in utero* development and positive energy balance are two such processes (Greene, 1939; Ingle, 1949; Mayer et al., 1954, 1956; Hill and Peters, 1998; Hill et al., 2003; Hill, 2006; Sun et al., 2011; Archer et al., 2013b, 2018; Archer, 2015a,b,c, 2018; Shook et al., 2015; Archer and McDonald, 2017), in this paper we extend our previous theoretic work, the *Maternal Resources Hypothesis* (Archer, 2015a,b,c,d; Archer and McDonald, 2017), by introducing two conceptual frameworks. The first, *Asymmetric Nutrient-Energy Partitioning* describes the context-dependent, cell-specific competition for calories that determines the partitioning of nutrient-energy to oxidation, anabolism, and/or storage. The second, *Effective Caloric Intake* describes the quantity of calories (i.e., nutrient-energy) available to constrain energy-intake via the inhibition of the sensorimotor cells that initiate ingestive behaviors (i.e., energy-sensing appetitive neuro-muscular networks in the liver and brain) (Langhans, 1996; Schwartz et al., 2000; Friedman, 2008; Allen et al., 2009; Woods, 2009). These frameworks are extensions of the ecological principles of exploitative and/or interference competition (Case and Gilpin, 1974; Weiner, 1990; Bourlot et al., 2014), and are founded upon well-established physiologic principles.

Briefly, we posit that the context-dependent inter-cellular competition for calories results in an a*symmetric nutrient-energy partitioning* that reduces the *effective caloric intake* of each meal. The relative lack of calories available to the energy-sensing, sensorimotor cells in the liver and brain initiates ingestive behaviors and energy intake. Inherent in this conceptualization is the independence and dissociation of the energetic demands of metabolism and the neuro-muscular networks that initiate ingestive behaviors and concomitant energy intake. The de-coupling of the initiation of ingestive behaviors from metabolic demands explains why individuals with substantial amounts of stored energy continue to chronically consume calories in excess of metabolic demands (i.e., over-nutrition).

While there are numerous phenomena that reduce *effective caloric intake* and lead to chronic increments in energy intake (e.g., exercise, puberty, and pregnancy), we posit that excessive fat-cell hyperplasia and physical inactivity are unique in that they unbalance metabolic-flux (i.e., the flow of nutrient-energy into and out cells) and by doing so, engender *false signals* of short-term energy homeostasis that cause more energy to be consumed and stored than expended. This leads to diminished insulin sensitivity, and increments in both body and fat mass, and metabolic diseases. Thus, our frameworks in concert with the *Maternal Resources Hypothesis* provide a parsimonious and physiologically rigorous explanation for the rapid rise in the global prevalence of increased body and fat mass, and/or metabolic dysfunction in humans and other mammalian species, inclusive of companion, laboratory, farm, and feral animals (Herberg and Coleman, 1977; Flather et al., 2009; Klimentidis et al., 2011; Ertelt et al., 2014; Hoenig, 2014; Sandoe et al., 2014; NEHS, 2015).

## The Conceptual Framework of Asymmetric Nutrient-Energy Partitioning

### Ecological Science

Competition is fundamental to the evolution of biological organisms (Darwin, 1859), and the asymmetric acquisition of energy and other resources via exploitative and interference competition are well-established phenomena (Case and Gilpin, 1974; Weiner, 1990; Bourlot et al., 2014). For example, in exploitation competition, organisms acquire and use (i.e., exploit) resources directly so that they are no longer available for use by other organisms. Thus, competitive advantages allow “*individuals* [to] *obtain a disproportionate share of the resources…and suppress the growth of smaller individuals*” (Weiner, 1990, p. 360). Given this foundation, our framework of *asymmetric nutrient-energy partitioning* extends the ecologic concept of resource competition from individual organisms to the inter-cellular competition for calories within the mammalian body.

To be precise, we do not use the competitive acquisition and exploitation of resources in the natural world as a mere analogy; rather, we posit that the cell-specific asymmetric competition and concomitant partitioning of nutrient-energy resources is central to understanding the rapid rise in global prevalence of obesity and metabolic disease in human and non-human animals. The essential element of this framework is the characterization of the mammalian body as an “ecosystem” in which disparate cell-types employ a diverse set of context-dependent competitive strategies to meet their unique demands for nutrient-energy.

### Body-as-Ecosystem and the Competition for Calories

We posit that the mammalian body is a complex, physiologic “ecosystem” in which survival and health are determined by metabolic-flux (i.e., the flow of energy through living cells) (Archer, 2015c, 2018; Archer and McDonald, 2017; Archer et al., 2018). An organism’s metabolic-flux is determined primarily by the energetic demands of the constituent populations of cells, energy-intake behaviors, and the availability of nutrient-energy to meet metabolic demands. Significant disturbances to metabolic-flux such as starvation (i.e., insufficient energy-intake relative to metabolic demands), exhaustion (i.e., excessive metabolic demands relative to energy intake), and physical inactivity (i.e., insufficient metabolic demands relative to energy intake) increase morbidity and mortality (Mayer, 1953; Mayer et al., 1954, 1956; Archer and Blair, 2011; Shook et al., 2015; Archer et al., 2017a, 2018; Archer, 2018).

Within each mammalian body, each cell must compete for nutrient-energy, and cell-types with competitive advantages will exploit (i.e., acquire, oxidize, and/or store) a greater percentage of consumed calories and serum energy substrates (e.g., glucose, amino and fatty acids) at the expense of less advantaged cell-types. As a result of the enhanced acquisition, advantaged cell-types (e.g., skeletal muscle- or fat-cells) will increase in size and/or number when possible. As discussed in subsequent sections, competitive strategies are context-dependent and therefore contingent upon inter- and extra-cellular environments [e.g., level of serum insulin and energy substrates, glycogen saturation and/or cellular 5^′^ adenosine monophosphate-activated protein kinase (Baron et al., 1988; Claret et al., 2007; Jensen et al., 2011; Friedrichsen et al., 2013; O’Neill, 2013)].

Furthermore, because the initial populations of the mammalian ecosystem (i.e., type, number, and quality of cells) are established during gestation, we posit that early development (e.g., *in utero* through puberty) is the critical period for the construction of the competitive milieu and concomitant partitioning of nutrient-energy that determine body mass and metabolic health trajectories from infancy to senescence (Archer, 2015c; Archer and McDonald, 2017).

#### Asymmetric Competition and Partitioning

The asymmetric inter-cellular competition and concomitant partitioning of nutrient-energy in mammals are well-established (Bauman and Currie, 1980; Bell et al., 1987; Ivy, 1987; Baron et al., 1988; Heymsfield et al., 2006; Halas et al., 2007; Thyfault et al., 2007; Peters, 2011). Yet with notable exceptions [e.g., see (Peters, 2011; Archer, 2015a,b,c,d; Archer and McDonald, 2017)], energy metabolism is not recognized as a cell- and context-specific competitive process. We argue that because competition is an essential feature of all levels of biology (i.e., from cells to societies), our conceptualization is essential for the understanding and treatment of obesity and energy-contingent metabolic diseases (e.g., type 2 diabetes mellitus, T2DM).

The asymmetric competition and partitioning of nutrient-energy in mammals follows from several physiologic facts. First, all living cells exhibit metabolic-flux, and therefore require energy intake to meet metabolic demands. Second, because the availability of nutrient-energy in the mammalian body is finite, undulating, and zero-sum (i.e., only one cell can dispose of any given molecule of an energy substrate), all cells compete to acquire the nutrient-energy necessary to meet their metabolic demands. Third, because cells differ in metabolic activity (Elia, 1992), context-dependent cell-specific strategies evolved to meet the unique energy demands of each cell type (e.g., insulin and contraction-mediated processes) (Ivy, 1987, 2004; Baron et al., 1988; DeFronzo, 1988; Ivy and Kuo, 1998; Jensen, 2003; Aas et al., 2005; Jensen and Richter, 2011; Peters, 2011). Fourth, because homeostasis and survival necessitated meeting the energy demands of *all cells*, the evolution of complex organisms, such as mammals required the development of sensorimotor (i.e., neuro-muscular) networks linking energy-sensing cells in the liver and brain to the musculoskeletal system. These networks maintain adequate levels of metabolic-flux by initiating energy-intake behaviors to meet chronic whole-body metabolic demands (i.e., the sum of cell-specific energy expenditures over time). Fifth, a large body of research demonstrates that chronic whole-body energy expenditure, which is comprised chiefly of the energetic demands of basal metabolism and physical activity, is the primary driver of habitual energy-intake (Mayer et al., 1954; Edholm et al., 1955; Blundell et al., 2003, 2015; Shook et al., 2015). Therefore, because both basal and physical activity energy expenditures are driven by cell-specific metabolic activity, it is logical to conclude that habitual energy-intake is driven primarily by the asymmetric, cell-specific *competition for calories* and the asymmetric partitioning of nutrient-energy to oxidation, anabolism, and/or storage.

Thus, we posit that in mammals there is an evolutionarily conserved relation between the sum of cell-specific metabolic activity over time (i.e., chronic basal and physical activity energy expenditure) and habitual energy intake. This relation was implied over two thousand years ago when Aristotle wrote that the defining characteristic of animals was the necessity of bodily movement (i.e., physical activity) in order to eat (i.e., energy intake), and contrasted the daily physical activity of animals with that of plants, which have the luxury of energy acquisition and survival despite stasis (Aristotle, 1943).

### Cell-Specific Competitive Strategies

While all living cells require and compete for nutrient-energy, herein we focus primarily on fat- and skeletal muscle-cells because these cell-types have the greatest influence on the competitive milieu and concomitant metabolic and health trajectories of the mammalian body. First, the defining characteristic of obesity is an excess of body-fat mass (Schwartz et al., 2017) attributable to a greater number and/or size of fat-cells (adipocytes) relative to other cell-types (e.g., myocytes) (Brook et al., 1972; Salans et al., 1973; Knittle et al., 1979; Sjostrom and William-Olsson, 1981). Second, unlike other cell-types, the storage of nutrient-energy in fat-cells is independent of their metabolic demands. Third, fat-cell plasticity (i.e., hypertrophic/hyperplastic potential) and capacity to store nutrient-energy is greater than other cell-types. Fourth, in healthy (i.e., physically active and insulin sensitive) individuals, the most successful competitors for serum glucose in the post-prandial period, and lipids in the post-absorptive period are skeletal muscle-cells (Baron et al., 1988; Dube et al., 2008; DeFronzo and Tripathy, 2009; Rabøl et al., 2011). Fifth, the competitive strategies and storage capacity of skeletal muscle-cells are variable and dependent on the chronic metabolic demands induced via physical activity (Ivy and Holloszy, 1981; Ivy, 1987; Jensen et al., 2011). Sixth, skeletal muscles-cell metabolism is a major determinant of resting energy expenditure (Zurlo et al., 1990) and in confluence with cardiac myocytes, is responsible for nearly 100% of physical activity energy expenditure. Seventh, alterations in the competitive advantages in skeletal muscle-cells (e.g., decrements in insulin sensitivity) are the major driver of metabolic diseases (DeFronzo, 1988; DeFronzo et al., 1992; DeFronzo and Tripathy, 2009). Finally, we posit that socio-environmental evolution over the past century induced the greatest phenotypic changes in fat- and skeletal muscle-cells compared with other cell-types (Archer and Blair, 2011; Church et al., 2011; Archer et al., 2013b,c, 2018; Archer, 2015a,b,c,d, 2018; Archer and McDonald, 2017).

### Competitive Advantages of Skeletal Muscle-Cells (Myocytes)

#### Insulin-Induced Competitive Advantages

Numerous context-dependent competitive strategies exist for the acquisition and storage of nutrient-energy in mammalian skeletal muscle-cells (myocytes) (Ivy, 1987; Baron et al., 1988; Jensen, 2003). For example, under hyperinsulinemia, the skeletal muscle-cells of insulin-sensitive individuals dispose of 70–90% of serum glucose at the expense of other tissues (Thiebaud et al., 1982; Baron et al., 1988; DeFronzo, 1988; Shulman et al., 1990). In fact, supra-physiologic doses of insulin (i.e., overdoses) induce such extreme competitive advantages in skeletal muscle-cells that neurons in the central nervous system cannot compete and are deprived of serum glucose. This exploitation competition results in neuroglycopenia, seizures, coma, and death (Cryer, 2007; Russell et al., 2009). Thus, insulin-dependent (i.e., context-specific) competitive advantages allow skeletal muscle-cells to exploit serum glucose at a rate that can deprive non-insulin dependent cells of nutrient-energy. In the context of starvation, this competitive advantage increases substantially (Goodman and Ruderman, 1979; Brady et al., 1981).

Conversely, the skeletal muscle-cells of insulin-resistant individuals fail to gain competitive advantages under hyperinsulinemia and dispose of significantly less serum glucose (DeFronzo, 1988; DeFronzo and Tripathy, 2009), thereby increasing the availability of glucose to other cell-types. These results demonstrate that the competitive advantages and partitioning of nutrient-energy to skeletal muscle-cells are context-dependent (e.g., serum insulin levels or dose of physical activity), and exemplify the ecological principles of exploitation and interference applied to the intercellular competition for nutrient-energy.

#### Physical Activity-Induced Competitive Advantages

Physical activity is the major modifiable determinant of the competitiveness and concomitant asymmetric partitioning of nutrient-energy to both hepatic (liver) and skeletal muscle-cells across mammalian species (Ivy and Holloszy, 1981; Ivy, 1987, 2004; Ivy and Kuo, 1998; Galassetti et al., 1999; Powell et al., 2002; Stewart-Hunt et al., 2006; Pratt et al., 2007; Krogh-Madsen et al., 2014). Specifically, physical activity induces contractions of skeletal muscle-cells that are metabolically costly and deplete stored nutrient-energy (e.g., glycogen and lipids) in a dose-dependent manner (i.e., frequency, intensity, duration, and mode/type of activity). The decrement in stored nutrient-energy causes increments in the uptake of serum glucose and lipids by skeletal muscle-cells via insulin-dependent and insulin-independent (e.g., contraction-induced) mechanisms (Ivy, 1987; Jensen, 2003). The increased disposal of serum glucose by skeletal muscle-cells stimulates hepatic-cell gluconeogenesis and glycogenolysis to maintain blood sugar levels. These endogenous glucose-producing processes are metabolically costly and reduce hepatic nutrient-energy stores (e.g., glycogen and lipids). Note: the metabolic costs of gluconeogenesis explain the effects of exercise on non-alcoholic fatty liver disease (Magkos, 2010; Chalasani et al., 2012).

Thus, the reductions in stored nutrient-energy due to physical activity lead to competitive advantages in both skeletal muscle- and hepatic-cells with concomitant increments in nutrient-energy disposal in these cells during the post-prandial and post-absorptive periods (Ivy and Holloszy, 1981; Ivy, 1987, 1991, 2004; Ren et al., 1994; Friedman, 1995; Perseghin et al., 1996; Boulé et al., 2001; Powell et al., 2002; Holloszy, 2005; Bergouignan et al., 2006, 2009, 2010, 2013, 2014; Stewart-Hunt et al., 2006; Thyfault et al., 2007; Dube et al., 2008; Krogh-Madsen et al., 2010; Jensen and Richter, 2011; Jensen et al., 2011; Thyfault and Krogh-Madsen, 2011; Davis et al., 2012; Friedrichsen et al., 2013; O’Neill, 2013). There is a great deal of heterogeneity in competitive strategies within skeletal muscle-cell sub-groups [e.g., glycolytic and oxidative (James et al., 1985)]; therefore, the effects of physical activity on metabolic-flux and partitioning of nutrient-energy to oxidation, anabolism, or storage will be dependent on the training status of the individual in concert with the dose of physical activity (i.e., frequency, intensity, duration, and mode).

All physical activity training protocols of sufficient dose induce competitive advantages and attendant increments in nutrient-energy disposal in both skeletal muscle- and hepatic-cells (Krogh-Madsen et al., 2014). Furthermore, exercise protocols of sufficient intensity and volume in concert with nutritional support (Phillips, 2011) (e.g., “bodybuilding” training) also induce skeletal muscle-cell hypertrophy and satellite cell activation with significant increments in nutrient-energy (e.g., amino acids, lipids, and glucose) disposal and oxidation, and metabolic control (Frontera et al., 1988; Phillips et al., 2005; Phillips, 2014; Snijders et al., 2015). Thus, exercise training increases the competitive advantages of skeletal muscle-cells via multiple mechanisms (e.g., insulin, contraction, and hypertrophic related processes). Therefore, both increased physical activity and greater muscle mass induce competitive advantages (Borghouts and Keizer, 2000; Boulé et al., 2003; Van Der Heijden et al., 2010; Srikanthan and Karlamangla, 2011; Shih and Kwok, 2018).

Conversely, physical inactivity (i.e., low whole-body metabolic-flux) causes dose-dependent decrements in hepatic- and skeletal muscle-cell metabolic-flux that drive decrements in the competitiveness of these cells via reductions in insulin sensitivity and total glycogen storage capacity (Ivy, 1991; Ren et al., 1994; Dube et al., 2008; Bergouignan et al., 2009, 2013; Krogh-Madsen et al., 2010; Jensen and Richter, 2011; Thyfault and Krogh-Madsen, 2011). Nevertheless, increases in physical activity restore the competitiveness of insulin resistant skeletal muscle cells (Devlin et al., 1987; Thyfault et al., 2007). Even a single bout of exercise is sufficient to enhance insulin sensitivity and the resultant competitiveness of skeletal muscle-cells while limiting the fuel available for increments in fat mass, hepatic and adipose tissue *de novo* lipogenesis, and ectopic fat deposition (Maehlum et al., 1978; Devlin et al., 1987; Larson-Meyer et al., 2006; Thyfault et al., 2007; Dube et al., 2008; Rabøl et al., 2011; Krogh-Madsen et al., 2014). Thus, the glycogen and lipid depletion-repletion cycles (i.e., metabolic-flux) induced via physical activity are essential for the maintenance of insulin sensitivity, metabolic flexibility [i.e., the ability to alter substrate oxidation as substrate availability changes (Galgani et al., 2008)], and concomitant metabolic health across species (Devlin et al., 1987; Ivy, 1987; Perseghin et al., 1996; Brooks, 1998; Ivy and Kuo, 1998; Powell et al., 2002; Stewart-Hunt et al., 2006; Pratt et al., 2007; Dube et al., 2008; Bergouignan et al., 2009, 2013; Jensen et al., 2011; Thyfault and Krogh-Madsen, 2011; Egan and Zierath, 2013; Friedrichsen et al., 2013; O’Neill, 2013; Hawley et al., 2014; Goodpaster and Sparks, 2017).

In summary, the ability of hepatic and skeletal muscle-cells to compete for nutrient-energy is dependent on metabolic-flux (i.e., substrate depletion-repletion cycles). Increments in physical activity induce dose-dependent competitive advantages, whereas physical inactivity decreases metabolic-flux, and concomitant nutrient-energy disposal. Thus, our framework of *asymmetric nutrient-energy partitioning* suggests that physical activity is the key to the prevention and treatment of metabolic dysfunction, and offers a comprehensive answer to the question of why physically active individuals exhibit a reduced risk of T2DM and other energy-contingent chronic non-communicable diseases (e.g., cardiovascular disease and non-alcoholic fatty liver disease) compared to inactive individuals (Irwin et al., 2003; Sigal et al., 2006; Hallsworth et al., 2011; Davis et al., 2012; Keating et al., 2012; Archer et al., 2013a; Fedewa et al., 2014; Krogh-Madsen et al., 2014).

### Competitive Strategies of Fat-Cells (Adipocytes)

The primary role of fat-cells in the mammalian “ecosystem” is to acquire and store nutrient-energy. While both fat- and skeletal muscle-cells use context-dependent competitive strategies such as insulin and gain substantial competitive advantages in the context of negative energy balance (Goodman and Ruderman, 1979; Arner et al., 1981; Brady et al., 1981), hyperplasia (i.e., increments in the number of a cell-type) is the main competitive strategy of fat-cells. Thus, *ceteris paribus*, the amount of energy partitioned to fat-cells will increase as a function of the number of fat-cells. This argument is supported by several facts. First, adipocyte number is the primary distinguishing feature of obesity across species (Brook et al., 1972; Salans et al., 1973; Knittle et al., 1979; Hager, 1981; Sjostrom and William-Olsson, 1981; Bjorntorp, 1996; Spalding et al., 2008; McLaughlin et al., 2014; Archer, 2015c). Second, a strong inverse relationship exists between the partitioning of dietary fat in obese versus lean humans and other mammals (Hocquette et al., 1999, 2007; Jackman et al., 2006; Westerterp, 2009). Third, increments and decrements in fat mass are functions of existing adiposity (i.e., fat-cell number and size) (Bell et al., 1987; Elia et al., 1999; Forbes, 2000; Kozusko, 2002). Fourth, early development is a major determinant of both fat-cell number and obesity (Spalding et al., 2008; Chandler-Laney et al., 2011; Adamo et al., 2012); and fifth, monozygotic twins concordant for birth weight exhibit similar fat-cell numbers, while in those discordant for birth weight, the smaller twin displays both lower body mass and fat-cell number (Ginsberg-Fellner, 1981).

Yet, the strongest evidence for the cellularity-based competitive strategy of adipocytes comes from experimental studies across species (Häger et al., 1978; Yukimura and Bray, 1978; Jackman et al., 2008). For example, after a dietary intervention in prepubescent girls, Häger et al. (1978) found that “*obese girls who were most successfully treated had the lowest increase in fat-cell number.”* In other words, greater increments in the number of fat-cells (i.e., hyperplasia) resulted in asymmetric competition and an increase in the partitioning of nutrient-energy to fat-cells with concomitant decrements in treatment success. Häger et al.’s (1978) findings were consistently replicated, and in a review of the literature, Arner and Spalding (2010) stated, *“hyperplastic obese individuals have a poorer treatment outcome following diet-induced weight loss than hypertrophic individuals…*.” Similarly, in rodents Jackman et al. (2008) found that fat-cell *“hyperplasia occurring early in relapse persists throughout the regain process and that the small, presumably new, adipocytes preferentially accumulate fat relative to their large adipocyte counterparts*.”

#### Mechanisms of Fat-Cell Hyperplasia

While a detailed discussion of the mechanisms of fat-cell hyperplasia is beyond the scope of this paper, it plays a pivotal role in the development of obesity and metabolic diseases. Hyperplasia results from the recruitment and differentiation of mesenchymal/progenitor cells and mitotic clonal expansion (Asakura et al., 2001; Tang et al., 2003; Laharrague and Casteilla, 2010; Shoham and Gefen, 2012; Tang and Lane, 2012; Gavin et al., 2016). Hyperplasia is both a normal component of fetal development (Archer, 2015c; Archer and McDonald, 2017), and a compensatory mechanism in response to chronic positive energy balance (i.e., overnutrition) (Archer et al., 2018). As described in subsequent sections, excessive fat-cell hyperplasia during gestation is principal phenomena leading to *inherited obesity* (Archer, 2015c; Archer and McDonald, 2017; Archer et al., 2018), whereas the hyperplasia induced via physical inactivity-induced positive energy balance (i.e., low metabolic-flux) is responsible for *acquired obesity*, increments in visceral adiposity, and ectopic fat deposition (Archer et al., 2018).

In our frameworks, ectopic fat deposition is a compensatory mechanism in response to the inability of skeletal muscle-cells and adipocytes within adipose tissue to dispose of excess serum lipids at the rate at which they are supplied via dietary fat consumption or *de novo* lipogenesis. As we posited previously, chronic positive energy balance at any point from gestation through senescence leads to a “*training effect for fat cell development*” (Archer, 2015b) because as existing fat-cells reach their hypertrophic potential (or maximum), hyperplasia is induced (Shoham and Gefen, 2012; Tang and Lane, 2012). Ectopic fat deposition is a serious manifestation because it exacerbates the competitive dominance of fat-cells by limiting the number of stem cells available for differentiation to muscle or bone while simultaneously increasing the number of fat-cells in non-adipose tissues.

In summary, a large body of prior research into hyperplastic obesity across species (Häger et al., 1978; Jackman et al., 2008) supports the hypothesis that increased adipocyte cellularity is the main competitive strategy of fat-cells, and that increments in adipocyte cellularity result in increasingly asymmetric and adipogenic nutrient-energy partitioning (Archer, 2015c,d; Archer and McDonald, 2017; Archer et al., 2018). Thus, for any given level of caloric intake, a larger number of fat-cells will acquire and sequester a larger percentage of total energy intake, leading to increments in both adiposity and body mass.

### Cooperative Strategies

The survival of complex social organisms (e.g., humans, canines, and rodents) required the evolution of both cooperative and competitive strategies at all levels of socio-biological organization (i.e., from cells to societies). In contrast to competitive strategies, cooperative mechanisms increase the availability of nutrient-energy to other cells, or at the societal level, conspecifics. For example, societal-level strategies allow the survival of the group by constraining dominant individuals from monopolizing nutrient-energy resources to the exclusion of conspecifics (e.g., preventing alpha males from consuming all available food). These cooperative strategies include both long and short term physiological signals (e.g., satiety hormones) such as leptin, cholecystokinin, and pancreatic poly-peptides (Austin and Marks, 2009) that cause decrements in ingestive behaviors as energy intake and energy storage increase.

At the cellular level, we posit that insulin resistance is the predominant cooperative strategy and operates by increasing the availability of serum glucose to other cells. For example, as the level of stored energy within hepatic- and skeletal muscle-cells increases (e.g., glycogen saturation and lipid accumulation), insulin sensitivity and the ability to store serum glucose as glycogen decline (Devlin et al., 1987; Ivy, 1987, 2004; Roden et al., 1996; Thyfault et al., 2007; Dube et al., 2008; Jensen et al., 2011). The concomitant reduction in the competitiveness of insulin-resistant cells increases the availability of nutrient-energy substrates to other cells, especially those that remain insulin sensitive.

Hepatic- and skeletal muscle-cell insulin resistance is induced in numerous contexts including the elevated levels of fatty-acid oxidation induced via hypo-caloric feeding, fasting, or starvation (Newman and Brodows, 1983; Björkman and Eriksson, 1985; Cigolini et al., 1985; Svanfeldt et al., 2003). This cooperative strategy diverts nutrient-energy substrates to other cells (e.g., neurons), and allows for the survival of all cells in the body. As we posited previously, the naturally occurring insulin resistance of pregnancy is a cooperative strategy that drives nutrient-energy to the fetus (Archer, 2015c; Archer and McDonald, 2017; Archer et al., 2018). Thus, in contrast to the current consensus on the pathological nature of insulin resistance, we posit that insulin resistance is an essential feature of mammalian metabolism, and our frameworks of competitive and cooperative strategies explain the evolutionary benefits of this cooperative strategy in the mammalian ecosystem. Nevertheless, in the context of elevated and incomplete fatty-acid oxidation due to obesity (Jensen et al., 1989; Kelley et al., 1999) or physical inactivity (Bergouignan et al., 2010, 2013, 2014), hepatic-insulin resistance leads to a decrement in metabolic flexibility with subsequent declines in metabolic health over time (Galgani et al., 2008; Goodpaster and Sparks, 2017).

With respect to fat-cell specific cooperative strategies, the nutrient-energy stored in fat-cells (e.g., glycerol and fatty-acids) is sequestered and not available to other cell-type until conditions of negative energy balance (e.g., fasting or elevated physical activity), hypoinsulinemia, and/or beta-adrenergic stimulation. Thus, akin to the competitive strategies, the cooperative strategies of fat-cells are also context-specific. Conversely, the nutrient-energy stored in skeletal muscle-cells as glycogen is never available to other cell-types due to the lack of glucose-6-phosphatase (i.e., the glycogen molecule is too large to leave the cell). Thus, the nutrient-energy partitioned to “selfish” skeletal muscle-cells is “lost” to other cells in the body and is not available to constrain ingestive behaviors. From an evolutionary perspective, the sequestering of nutrient-energy in mammalian skeletal muscle-cells is adaptive given the necessity of physical activity for the survival of both the individual and the species (e.g., fight-flight and mating behaviors, acquisition of nutrient-energy).

In the following section, we introduce the conceptual framework of *effective caloric intake* to explain how the asymmetric inter-cellular competition and partitioning of nutrient-energy drives increments in ingestive behaviors and energy intake.

## The Conceptual Framework of Effective Caloric Intake

The framework of *effective caloric intake* describes the amount of nutrient-energy available to constrain energy-intake via the inhibition of the sensorimotor cells that govern ingestive behaviors (i.e., energy-sensing appetitive neuro-muscular networks in the liver and brain) (Langhans, 1996; Schwartz et al., 2000; Friedman, 2008; Allen et al., 2009; Woods, 2009). We posit that the availability of nutrient-energy to each cell is constrained not only by ingestive behaviors and total energy-intake, but also by the context-dependent, asymmetric competition between individual cells. Thus, when energy-sensing, appetitive cells in the liver and brain are “outcompeted” by other cell-types (e.g., fat and/or muscle-cells), the *effective caloric intake* of a meal is diminished, and total energy-intake will be increased to compensate for the deficit (Archer, 2015c, 2018; Archer and McDonald, 2017; Archer et al., 2018).

Inherent in this framework is the independence and dissociation of the energetic demands of metabolism and the sensorimotor (i.e., neuro-muscular) pathways that initiate ingestive behaviors. We posit that eating and drinking can be stimulated by either *real* or *apparent* deficits in energy homeostasis (i.e., true or false signals). Thus, when ingestive behaviors are stimulated by *real* deficits, such as those induced by starvation or increments in physical activity, energy-intake will be increased to maintain homeostasis and ensure survival. In contrast, when ingestive behaviors are chronically initiated via merely *apparent* deficits in energy homeostasis (i.e., false signals) induced via excessive fat-cell hyperplasia or physical inactivity (i.e., low metabolic flux), energy-intake is increased above metabolic demands, leading to positive energy balance, *acquired obesity*, and/or metabolic diseases (Archer, 2015c, 2018; Shook et al., 2015; Archer and McDonald, 2017; Archer et al., 2018).

The independence and dissociation of deficits in energy homeostasis and the initiation of ingestive behaviors can be illustrated by a simple example. If after fasting for 48 h, you find several large insects crawling in your food, your ingestive behaviors and energy intake will be diminished while your body’s metabolic demand for nutrient-energy is unaffected. In time, the deficit in nutrient-energy sensed by the sensorimotor appetitive cells in the liver and brain will result in renewed eating and drinking. There is a large body of literature delineating the dissociation of ingestive behaviors and nutrient-energy surpluses and deficits (Leibowitz et al., 1981; Elmquist et al., 1999). We think the failure to distinguish these processes and speculations based of non-observable phenomena (i.e., mental states; e.g., appetites, perceptions, drives, needs, wants, etc.) contributes to the current confusion and lack of progress surrounding obesity and metabolic diseases (Archer et al., 2018).

### Physical Activity, Fat-Cell Hyperplasia, and *Effective Caloric Intake*

Physical activity and fat-cell hyperplasia reduce the *effective caloric intake* of meals because both lead to a reduction in the nutrient-energy available to inhibit the sensorimotor appetitive cells in the liver and brain that govern ingestive behaviors. For example, as explained previously, physical activity induces skeletal muscle-cells to exploit (i.e., acquire and use) nutrient-energy at the expense of other less-advantaged tissues (e.g., adipocytes, neurons, and hepatocytes). Thus, because the nutrient-energy partitioned to skeletal muscle-cells is not available to constrain ingestive behaviors, total energy-intake will be increased to compensate for the *real* deficit in energy homeostasis. Therefore, reductions in *effective caloric intake* from physical activity lead to an increase in eating and drinking and a *necessary* increment in total energy-intake over time.

Thus, decrements in *effective caloric intake* from physical activity provide a true signal of deficits in energy homeostasis; and because physical activity induced increments in energy-intake are driven in parallel with dose-dependent increases in skeletal muscle-cell energy expenditure, the overall effect on whole body energy-balance is neutral. As such, and as explained in detail in a later section, increments in exercise or physical activity in active (i.e., non-sedentary) individuals will not lead to significant long-term weight-loss because body mass will be maintained at a higher level of metabolic-flux (i.e., greater caloric intake and energy expenditure).

Conversely, increments in fat-cell hyperplasia lead to reductions in *effective caloric intake* and increments in energy-intake that are not matched by parallel increases in energy expenditure. This occurs because, in contrast to muscle-cells, the competitive advantages and storage capacity of fat-cells are driven by number and size, and not their metabolic demands. As stated, a larger number of fat-cells will acquire and store a larger percentage of total energy-intake independent of their metabolic demands. Therefore, in contrast to physical activity, increments in fat-cell hyperplasia lead to *apparent* deficits (i.e., *false signals*) in short-term energy homeostasis that cause more energy to be consumed and stored than expended. This leads to increments in both body and fat mass with concomitant weight-dependent decrements in physical activity and insulin sensitivity. Our arguments are supported by research demonstrating that appetitive processes are more sensitive to stimuli from nutrient-energy metabolism and related hormones and cytokines than total fat mass *per se* (e.g., serum glucose, insulin, leptin, hepatic metabolic-flux and adenosine triphosphate/adenosine diphosphate ratio, gut peptides) (Woods et al., 1985; Langhans, 1996; Friedman et al., 1999; Schwartz et al., 2000; Friedman, 2008; Allen et al., 2009; Woods, 2009).

In summary, physical activity and fat-cell hyperplasia lead to the asymmetric competition and partitioning of nutrient-energy to skeletal muscle- and fat-cells, respectively. The disproportionate disposal of nutrient-energy reduces the *effective caloric intake* of each meal by lessening the absolute amount of nutrient-energy available to inhibit the sensorimotor cells in the liver and brain that govern ingestive behaviors. This reduction in available energy leads to compensatory increases in energy-intake to overcome *real* or merely *apparent* deficits. While physical activity engenders a *real* perturbation in energy homeostasis (i.e., a *true signal)* that necessitates an increase in total energy-intake to ensure survival, excessive fat-cell hyperplasia leads to an *apparent* deficit (i.e., *false signal*) that drives increments in ingestive behaviors and the overconsumption of calories. This leads to chronic positive energy balance and subsequent increments in body and fat mass, and metabolic diseases. Thus, an individual’s nutrient-energy consumption over time will equal or exceed the sum of chronic cell-specific metabolic activity (i.e., basal and physical activity energy expenditure) plus the nutrient-energy sequestered in fat-cells and other low metabolically active tissues.

To be precise, we do not argue that decrements in *effective caloric intake* are the *only* mechanism that drives energy intake. However, we argue that the asymmetric competition for nutrient-energy and concomitant reductions in the inhibition of energy-sensing appetitive cells in the liver and brain are the *primary drivers* of habitual energy-intake above basal metabolic energy requirements.

## The Etiologies of Inherited and Acquired Obesity

In the following sections, we provide empirical support for our hypothesis that the habitual overconsumption of nutrient-energy and concomitant elevated serum energy substrates (e.g., glucose, fatty-acids, and cholesterol) characteristic of both *inherited* and *acquired obesity* and metabolic diseases (e.g., T2DM) are caused by the *asymmetric nutrient-energy partitioning* driven primarily via *in utero* engendered increments in fat- and beta-cell hyperplasia and/or physical inactivity induced decrements in metabolic-flux.

### Inherited Obesity: *Accumulative Maternal Effects* and the *Maternal Resources Hypothesis*

In the *Maternal Resources Hypothesis* (Archer, 2015a,b,c,d; Archer and McDonald, 2017), we posited that *inherited obesity* was the result of the irreversible competitive dominance of fat-cells engendered during gestation via non-genetic evolutionary processes known as *accumulative maternal effects* (Archer, 2015c; Archer and McDonald, 2017). Briefly, we argued that the rapid rise in the population prevalence of increased body and fat mass, and metabolic dysfunction in the latter half of the 20th century were engendered by the effects of socio-environmental evolution over the past century (e.g., reduced pathogenic load, decreased physical activity, and improved nutrition) (Church et al., 2011; Archer et al., 2013b,c). These phenomena led to cumulative increments in maternal energy resources (i.e., body mass and adiposity) and decrements in maternal physical activity. When a mother’s physical activity fell below her *“Metabolic Tipping Point”* (Archer et al., 2018), the competitive advantages of her skeletal muscle-cells were reduced. This decrement altered the competitive relation between mother and conceptus thereby increasing the availability of nutrient-energy to the fetus. This fetal “over-nutrition” induced several allometric, physiologic, and behavioral inheritances that irreversibly engendered a competitive dominance of fat-cells for the lifespan of the offspring (Archer, 2015c).

First, the excess nutrient-energy caused increments in fetal cellularity (Szabo and Szabo, 1974; Kalkhoff, 1991; Catalano and Hauguel-De Mouzon, 2011), with disproportionate increments in fetal fat- and pancreatic beta-cells (Szabo and Szabo, 1974; Kervran et al., 1978; Kalkhoff, 1991; Herrera and Amusquivar, 2000; Martens and Pipeleers, 2009; Chandler-Laney et al., 2011; Portha et al., 2011; Long et al., 2012) while negatively altering skeletal muscle-cell development (e.g., decreased contractile proteins with increased collagen accumulation and crosslinking) (Tong et al., 2009; Huang et al., 2012). These latter alterations permanently reduced the competitiveness of skeletal muscle-cells by altering the quality of fetal skeletal muscle-cells (e.g., force production), leading to decrements in physical activity and cardio-respiratory fitness from infancy to adulthood. These latter arguments have strong support (Tomkinson et al., 2003, 2012; Olds et al., 2006; Malina and Little, 2008; Archer and Blair, 2011; Church et al., 2011; Archer et al., 2013b,c, 2017a).

Second, the intensified insulin response (via enhanced beta-cell mass and function) and fat-cell hyperplasia in concert with reduced competition from inactive and dysfunctional skeletal muscle-cells exponentially increased the asymmetric partitioning and sequestering of nutrient-energy in fat-cells (i.e., a larger number of fat-cells acquiring and storing a larger percentage of total energy intake). The asymmetric partitioning of nutrient-energy to storage in fat-cells reduced the *effective caloric intake* of meals and drove increased energy intake, positive energy balance, and increments in both body mass and adiposity. The increased body mass further reduced physical activity via diminished strength-to-weight ratio [i.e., bigger or weaker individuals move less than smaller or stronger (Chirico and Stunkard, 1960; Archer et al., 2013a)] with additional decrements in the competitiveness of skeletal muscle-cells. These permanent and irreversible allometric, physiologic, and behavioral alterations were critical to the etiology of the inherited (i.e., childhood) obesity epidemic (Archer, 2015c; Archer and McDonald, 2017; Archer et al., 2018).

#### Racial and Socio-Economic Disparities in Obesity

The *Maternal Resources Hypothesis* suggests that disparities in obesity in the US that vary by race and socio-economic status are in fact driven by differences in matrilineal pre-conception and pre-natal physical activity, body cellularity, and metabolic-flux (Archer, 2015c,d; Archer and McDonald, 2017; Archer et al., 2018). For example, black girls decrease their physical activity to a greater degree than white girls during adolescence (Kimm et al., 2002) and black mothers as a group are less physically active, less affluent, less well-educated (Evenson et al., 2004; Schmidt et al., 2006; Haakstad et al., 2007; Archer et al., 2013b,c; Most et al., 2018), and have children with greater adiposity and risk of metabolic diseases (Archer, 2015c; Archer and McDonald, 2017). Thus, what appears to be genetic or socially mediated processes, are in fact driven by non-genetic evolutionary processes induced via low levels of matrilineal/maternal physical activity and concomitant loss of metabolic control and overconsumption.

#### Other Developmental Pathologies Related to Maternal Effects

There are numerous developmental pathologies (e.g., increased fetal mortality, congenital deformities, low birth-weight, and reduced neonate survival) that we posit are caused by the negative effects of excessive fat-cell mass and the overconsumption of nutrient-energy induced by low-levels of physical activity and concomitant low skeletal muscle- and hepatic-cell metabolic-flux (Archer et al., 2018). For example, the physical space constraints and increments in intrathoracic pressure engendered via excessive fat-cell mass (i.e., fat mass compressing the uterus, placenta, and/or supporting vasculature) and sedentary behavior reduce blood flow to both the placenta and fetus (i.e., placental and fetal ischemia). It was demonstrated that both higher body mass and sitting generated greater intraabdominal pressure (Cobb et al., 2005) and increased pressure correlated with comorbidities (Sugerman et al., 1997). Thus, we posit that a portion of obese, sedentary mothers risk “starving” their fetuses of both oxygen and nutrient-energy.

Similarly, if pregnancy fails to induce sufficient increments in nutrient-energy intake, the fetus may be spontaneously aborted, exhibit intrauterine growth restriction and/or developmental defects. The mechanism is simple; if the naturally occurring insulin resistance of pregnancy is over-whelmed by the preexisting insulin resistance and poor metabolic control induced via low physical activity, low metabolic-flux, and excessive fatty acid oxidation (Archer, 2015c;Archer and McDonald, 2017; Archer et al., 2018), ingestive behaviors and energy intake will not be stimulated in parallel with the increased nutrient-energy demands of fetal development. Thus, the fetus receives an inadequate nutrient supply because the mother’s cells outcompete the placenta and fetus’ ability to compensate. This conceptualization is supported by a large body of experimental evidence demonstrating that restrictions of the nutrient supply to the intrauterine milieu induce numerous fetal pathologies (Wallace, 2000; Hay, 2006; Long et al., 2012; Limesand et al., 2013).

Thus, alterations in the competitive milieu (e.g., over-nourished or restricted) may explain the high rate of negative birth outcomes in populations that experience low physical activity, low metabolic flux, and high levels of obesity (e.g., African-American women) (Hogue and Hargraves, 1995; Lu and Halfon, 2003). Evidence suggest that these pathologies occur in populations with similar socio-economic status (Schoendorf et al., 1992), medical care (Barfield et al., 1996), and use of assisted reproductive therapies (Feinberg et al., 2006; Seifer et al., 2008; Mukherjee et al., 2013). Therefore, we contend that disparities in obesity, metabolic diseases, and birth outcomes are not driven primarily by genetic or socio-economic factors (Archer et al., 2018).

#### Iatrogenic Artificial Selection

In the *Maternal Resources Hypothesis*, we posited that the increased use of Cesarean sections over multiple generations led to the artificial selection for progressively larger, increasingly physically inactive, and metabolically compromised offspring predisposed to obesity and metabolic diseases (Archer, 2015c; Archer and McDonald, 2017). Prior to the 20th century, morbid and super-morbid obese individuals were extremely rare, in part because macrosomic fetuses and their metabolically compromised mothers were subject to greater selection pressures (e.g., suffocation and hemorrhage) from cephalo-pelvic disproportion (i.e., incongruity of fetal head size and birth canal capacity) (Wells et al., 2012). Stated simply, the fetuses were too large to exit the birth canal. Thus, advances in obstetric care over the past century were a primary driver of increments in the frequency of larger humans in general but especially obese, inactive, metabolically compromised phenotypes in populations that had access to medicalized childbirth over multiple generations.

The strongest evidence for this iatrogenic artificial selection is the extremely rapid and disproportionate increases in severe and morbid obesity (i.e., Class II and III) among children and adolescents (Skelton et al., 2009), and adults varying by race, sex, and socio-environmental contexts (Sturm, 2007; Skelton et al., 2009). The *Maternal Resources Hypothesis* is the only theory that offers a mechanistic and physiologically rigorous explanation for the population shifts in both body and fat mass distribution.

#### Accumulative Maternal Effects in Non-human Animals

While the *Maternal Resources Hypothesis* focused principally on the inheritance of obesity in humans (Archer, 2015c; Archer and McDonald, 2017), the non-genetic evolutionary mechanisms of *accumulative maternal effects* are applicable to all mammals that experienced a recent, rapid rise in the prevalence of increased body mass, adiposity, and/or metabolic dysfunction (Archer and McDonald, 2017; Archer et al., 2018) [e.g., dogs, cats, horses, rodents, monkeys, deer, and elk (Herberg and Coleman, 1977; Flather et al., 2009; Klimentidis et al., 2011; Ertelt et al., 2014; Hoenig, 2014; Sandoe et al., 2014; NEHS, 2015; Montoya-Alonso et al., 2017)]. *Accumulative maternal effects* in mammals for body cellularity (e.g., adiposity), body mass, behavior (e.g., physical activity), and/or risk of disease were demonstrated consistently over the last century. In the 1930s, Walton and Hammond (1938) demonstrated unequivocal “maternal effects” for growth and body mass in horses and ponies, and in the 1950s Falconer demonstrated *accumulative maternal effects* for cellularity, body mass, and disease in mice (Falconer, 1965, 1967, 1973; Falconer et al., 1978). These non-genetic effects were replicated repeatedly across species (Rossiter, 1996; Fox and Mousseau, 1998; Mousseau and Fox, 1998; Bonduriansky and Day, 2009; Mousseau et al., 2009) inclusive of rodents (Garg et al., 2013), chickens (Liu et al., 1993), sheep (Maria et al., 1993), free-ranging cervids (Freeman et al., 2013), horses (Allen et al., 2002, 2004), and humans (Brooks et al., 1995; Archer, 2015c; Archer and McDonald, 2017).

Given the parallel increases in body mass, adiposity, and/or metabolic disease across diverse species existing in disparate environments, current anthropocentric theories positing diet-centric influences (Bray et al., 2004; Swinburn et al., 2011), thrifty genes (Neel, 1962) and predation release (Speakman, 2007) in the etiology of obesity and metabolic disease are inadequate. Thus, our frameworks in confluence with the *Maternal Resources Hypothesis* subsume or refute dietary and genetic speculations and provide a comprehensive and mechanistic evolutionary theory that explains the inheritance and familial resemblance for body mass, adiposity, physical activity, and metabolic dysfunction in both human and non-human animals.

### Obesity: A Homeorhetic Condition

**FIGURE 1:**
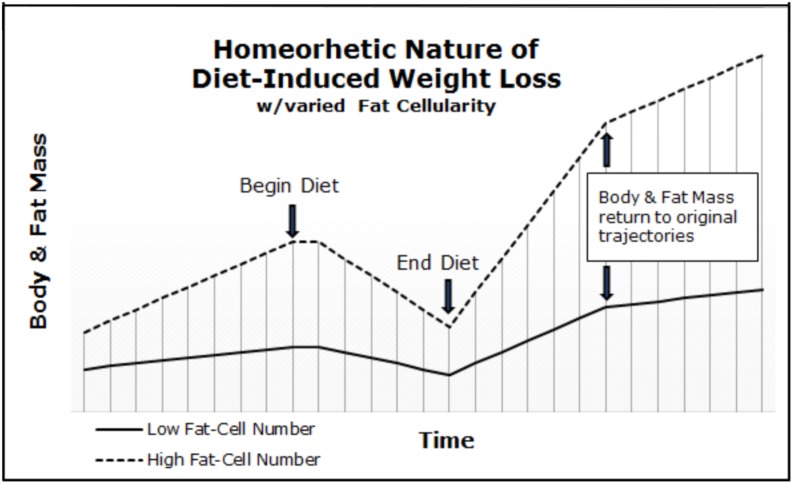
Body and fat mass trajectories of individuals varying in adipocyte cellularity. Body and fat mass trajectories return to the initial slope (i.e., rate of gain) after diet-induced weight loss. The initial slope was determined by fat-cell number.

**FIGURE 2:**
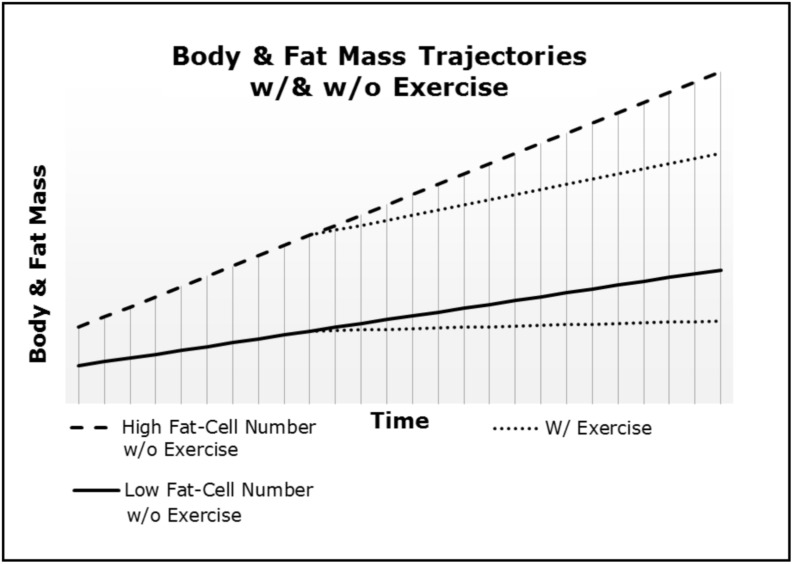
Body and fat mass trajectories (i.e., rate of gain) differ as a function of both fat-cell number and exercise.

Our frameworks in concert with the *Maternal Resources Hypothesis* explain why *inherited obesity* is a homeorhetic and not a homeostatic condition. As depicted in **Figure 1**, after a dietary or “lifestyle” intervention the body and fat mass quickly return to the initial trajectory engendered via the fat-cellularity created during gestation and early development (i.e., *in utero* through puberty) (Archer, 2015c; Archer and McDonald, 2017). Thus, body and fat mass trajectories are neither a set-point, nor a settling point and the slope will be determined by the initial populations of cell-types (e.g., ratio of fat-cells to skeletal muscle cells) and physical activity. Importantly, our frameworks explain the often-insurmountable physiological barriers to long-term weight loss in those suffering from morbid or severe obesity. First, the negative energy balance induced via hypocaloric dieting and/or exercise merely reduces the stored energy and size of fat-cells without altering the competitive advantage of increased fat-cell number. Second, smaller fat-cells have a competitive advantage over larger fat-cells (e.g., greater surface area to volume ratio). Third, as depicted in **Figure 2**, exercise merely decreases the slope of body and fat mass trajectories (i.e., rate of gain). Therefore, greater adipocyte cellularity increases the rate of both weight gain and regain (as per **Figure 1**), and increases the effort required to achieve and maintain weight loss (as per **Figure 2**). Thus, both fat-cell number and the physical activity-induced metabolic demands of skeletal muscle-cells determine the percentage of calories partitioned to fat- and muscle-cells, respectively. The reduction in the slope is why exercise ameliorates the regain of both fat and body mass after hypo-caloric interventions when compared to diet-alone.

Thus, our frameworks explain why the majority of obese children become obese adolescents and adults, and why greater than 95% of individuals suffering from inherited obesity fail to achieve and maintain a body-mass index (BMI) under 30 (Fildes et al., 2015), despite extreme interventions (Fothergill et al., 2016).

In summary, we posited that the fat and body mass trajectories of *inherited obesity* were engendered by *accumulative maternal effects* leading to metabolically compromised human (and non-human) infants with intensified insulin secretion, excessive adipocyte cellularity, and dysfunctional muscle-cell development predisposing to physical inactivity. These alterations irreversibly altered the competitive milieu of the body and permanently established the dominance of fat-cells in the acquisition, storage, and sequestering of nutrient-energy.

### The Etiology of Acquired Obesity

*Acquired obesity* is the excessive hypertrophy of existing fat-cells and recruitment of new fat-cells (hyperplasia) driven by the chronic positive energy balance induced by physical inactivity. This differs from *inherited obesity* in which the disturbance in metabolic-flux is driven primarily by fat and beta-cell hyperplasia and dysfunctional skeletal muscle-cells engendered *in utero* via non-genetic evolutionary forces (i.e., *accumulative maternal effects*) (Archer, 2015a,b,c; Archer and McDonald, 2017; Archer et al., 2018). As explained below, in contrast to the rapidity with which *inherited obesity* develops (i.e., 9 months of gestation), *acquired obesity* is incrementally instantiated over years and decades.

#### Physical Inactivity, Low Metabolic-Flux, and the “Metabolic Tipping Point”

**FIGURE 3:**
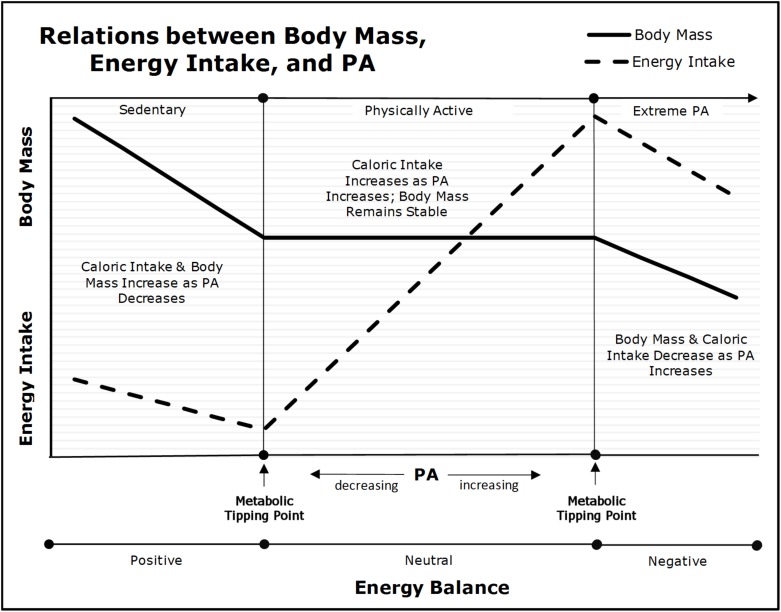
Relations between physical activity (PA), body mass, and energy intake (adapted from Archer, 2018). As physical activity declines below the “Metabolic Tipping Point” (i.e., into the “Sedentary” range), energy intake and energy expenditure become dissociated due to insufficient metabolic-flux, and as a result, body mass will begin to increase as energy balance becomes positive.

In the 1950s, it was established experimentally and observationally in rodents and humans (Mayer, 1953; Mayer et al., 1954, 1956), that the inter-relations between changes in body mass, energy intake, and physical activity were curvilinear. These results were replicated in a variety of settings (Stubbs et al., 2004; Shook et al., 2015). As shown in the center of **Figure 3**, there is a range of physical activity (denoted as “Physically Active”), in which habitual appetitive processes and energy-intake parallel physical activity energy expenditure such that body mass is maintained (Mayer, 1953; Mayer et al., 1954, 1956; Shook et al., 2015). For example, as Beaulieu et al. (2017) found, “*higher habitual PA* [physical activity] *improves acute homeostatic appetite control*.” Our frameworks render the underlying mechanisms of these results unambiguous. As explained previously, increments in physical activity induce dose-dependent competitive advantages that allow skeletal muscle-cells to exploit nutrient-energy resources at the expense of less advantaged cells. This asymmetric partitioning leads to reductions in the *effective caloric intake* of subsequent meals with concomitant increments in energy-intake via reductions in inter-meal periods and/or increased energy density per meal. Yet, despite the increased energy intake, body mass remains stable across a wide-range of doses of physical activity because the increased skeletal muscle-cell energy expenditures are compensated by parallel increments in energy intake. Thus, increments in physical activity merely lead to greater metabolic-flux with no changes in body mass. Importantly, if the increment in physical activity is large, basal energy expenditures will decrease because less energy is available to non-skeletal muscles cells (e.g., neurons in the central nervous system) inducing reductions in basal metabolic demands. These decrements in basal energy expenditures reduce the increment in energy-intake necessary to maintain energy homeostasis and body mass (Westerterp, 1998; Speakman and Selman, 2003; Westerterp and Plasqui, 2004).

Nevertheless, as depicted on the left side of **Figure 3** when an individual’s physical activity falls below their “Metabolic Tipping Point” (Archer, 2015c, 2018; Archer and McDonald, 2017; Archer et al., 2018) (denoted as “Sedentary”), energy-intake becomes dissociated from energy expenditure (Mayer, 1953; Mayer et al., 1954, 1956; Stubbs et al., 2004; Shook et al., 2015; Beaulieu et al., 2017). As Westerterp and Plasqui (2009) stated, *“The change from a physically active to a more sedentary routine does not induce an equivalent reduction of energy intake.”* Over time, small increments in energy-intake coupled with low physical activity lead to gains in fat mass and concomitant decrements in insulin sensitivity. The increased body and fat mass lead to further declines in physical activity from reduced strength-to-weight ratio [i.e., bigger or weaker individuals move less than smaller or stronger (Chirico and Stunkard, 1960; Archer et al., 2013a)]. The lack of hepatic- and skeletal muscle-cell metabolic-flux induced via physical inactivity initiates a cascade of metabolic dysfunction that drives both peripheral and central insulin resistance, positive energy balance, and increments in both fat-cell number and size. Over time, these physical inactivity-induced phenomena lead to *acquired obesity* and/or metabolic disease (e.g., T2DM) (Archer, 2015c; Archer et al., 2017a, 2018; Archer and McDonald, 2017). Thus, there is a minimum amount of physical activity-induced hepatic and skeletal muscle-cell metabolic-flux (i.e., substrate depletion-repletion cycles) necessary to maintain energy homeostasis and metabolic health.

It is important to note that if the chronic positive energy balance and reduced metabolic-flux characteristic of *acquired obesity* continues over time, existing fat-cells eventually reach their hypertrophic potential (or maximum) and there will be a *“training effect for fat cell development”* (Archer, 2015b) via the enhanced recruitment of mesenchymal cells (Sjostrom and William-Olsson, 1981; Archer and McDonald, 2017). The resulting fat-cell hyperplasia renders the distinction between *inherited* and *acquired obesity* after sexual maturity equivocal.

#### Individual Differences

Individuals vary in both inherited and acquired ratios of skeletal muscle-cells to fat-cells. For example, during pubescence, sexual dimorphism in adipogenesis and shifts in the ratio of skeletal muscle-cell to fat-cell mass are well established. In humans, as body-cell mass is increased, males gain a greater proportion of skeletal muscle-cell mass relative to fat-cell mass, whereas females exhibit the converse (Butte et al., 2007). In adulthood, it is well-established that increments in fat mass are a function of the individual’s existing adiposity (Bell et al., 1987; Elia et al., 1999; Forbes, 2000; Kozusko, 2002). Thus, the amount of physical activity necessary to inhibit chronic positive energy balance and the asymmetric partitioning of nutrient-energy to storage in fat-cells varies. Individuals with low fat-cell numbers or high skeletal muscle- to fat-cell ratio (e.g., lean muscular males) require less physical activity to maintain metabolic-flux and offset adipogenic nutrient partitioning and weight gain (see **Figure 2**). Individuals born with an excessive number of fat cells relative to skeletal muscle-cells will need a dose of physical activity that may be beyond their capacity. For these individuals, increments in body and fat mass are inevitable because as energy-intake is stimulated by physical activity induced decrements in *effective caloric intake*, the larger number of fat-cells “outcompete” other cell-types and sequester a larger amount of total energy intake. This conceptualization provides a rigorous, mechanistic explanation for the ubiquitous failure of non-surgical treatments of obesity. Nevertheless, at some point, the absolute caloric intake is sufficient to inhibit the sensorimotor appetitive cells and constrain energy intake so that body and fat mass stabilize at a new, higher level.

In summary, as individuals reduce their physical activity below their “Metabolic Tipping Point” (Archer et al., 2018), reductions in both hepatic and skeletal muscle-cell metabolic-flux engender positive feedback loops that lead to increments in energy intake, chronic positive energy balance, and the asymmetric competition and partitioning of nutrient-energy. The ensuing “*high storage but low triglyceride removal promotes fat tissue accumulation and obesity*” (Arner et al., 2011).

## Type-Ii Diabetes Mellitus: Diminished Metabolic-Flux and the Competitive Failures of Skeletal Muscle and Fat-Cells

A large body of research over the past 50 years demonstrates that the *“primary defect”* (DeFronzo and Tripathy, 2009) driving T2DM is a decrement in skeletal muscle-cell insulin sensitivity leading to both peripheral and central insulin resistance (DeFronzo, 1988; Shulman et al., 1990; DeFronzo et al., 1992; Holloszy, 2005; DeFronzo and Tripathy, 2009). While these findings suggest that T2DM is simply the result of the inability of skeletal muscle-cells to dispose of excess serum glucose, our frameworks instruct otherwise. Since all cell-types compete for nutrient-energy and the main function of fat-cells is the storage of excess energy, T2DM can be most precisely characterized as the failure of both skeletal muscle- and fat-cells to compete for and dispose of the nutrient-energy consumed in excess of metabolic demands.

When physical activity falls below the “Sedentary” metabolic tipping point (as per **Figure 3**), energy intake begins to increase despite the decreasing energy expenditure (Mayer, 1953; Mayer et al., 1954, 1956; Shook et al., 2015). We posit this occurs because as hepatic-cell metabolic-flux declines, these cells become saturated with glycogen and metabolites from fatty-acid oxidation (Koves et al., 2008). This leads to decrements in insulin sensitivity and metabolic flexibility [i.e., the ability to alter substrate oxidation as substrate availability changes (Kelley et al., 1999; Galgani et al., 2008; Bergouignan et al., 2011; Goodpaster and Sparks, 2017)]. As discussed previously, the only contexts in mammalian evolutionary history in which hepatic-cells experienced elevated levels of fatty-acid oxidation would be starvation and/or chronic elevated physical activity. Given the fact that these contexts induce the initiation of ingestive behaviors and energy intake and a reduction in basal energy expenditure to ensure survival, we posit that physical inactivity (i.e., low metabolic-flux) provides a *false signal* that drives increments in energy intake with concomitant decrements in energy expenditure (Mayer, 1953; Mayer et al., 1954; Stubbs et al., 2004; Shook et al., 2015). The positive energy balance increases the availability of serum energy substrates for hepatic and adipose tissue *de novo* lipogenesis, increments in fat-cell mass, and ectopic fat deposition (Strawford et al., 2004; Larson-Meyer et al., 2006; Dube et al., 2008; Rabøl et al., 2011).

Nevertheless, as long as the capacity to expand fat-cell mass and/or recruit new, smaller adipocytes from the mesenchymal stem-cell pool is maintained, whole body insulin sensitivity will not decline significantly. Thus, fat-cell hyperplasia and hypertrophy allow for the disposal of excess serum glucose and lipids (Heilbronn et al., 2004; Roberts et al., 2009). This compensatory mechanism allows individuals to remain metabolically healthy despite increasing body and fat mass. This conceptualization suggests that skeletal muscle- and fat-cells act as energy “sinks” that prevent the increase in serum energy substrates that lead to metabolic diseases such as T2DM. Nevertheless, because increments in body mass reduce physical activity [i.e., heavier individuals move less than lighter individuals (Chirico and Stunkard, 1960; Archer et al., 2013a; Archer and McDonald, 2017)], many “metabolically healthy but obese” individuals (Mathew et al., 2016) will progress toward metabolic disease (Short and Joyner, 2002; Soriguer et al., 2013) as physical activity declines over time. This is especially true with older individuals whose strength-to-weight ratio is already in decline (McGlory et al., 2017). Thus, when individuals are physically inactive and have limited fat-cell plasticity, serum energy substrates rise over time. This occurs because as physical inactivity drives increased energy-intake in concert with decrements in skeletal muscle-cell insulin sensitivity, the ability of pancreatic beta-cells to compensate for the reduced disposal of serum glucose declines. Over time, this leads to T2DM as pancreatic-beta cells become overloaded/exhausted and/or lose their sensitivity to serum glucose (DeFronzo, 1988; DeFronzo et al., 1992; DeFronzo and Tripathy, 2009).

In summary, our frameworks and existing evidence suggest that there is a minimum amount of physical activity metabolic-flux (e.g., glycogen and lipid depletion-repletion cycles) that is necessary to maintain energy homeostasis and prevent *acquired obesity* and metabolic diseases (Colberg et al., 2016). In conclusion, T2DM is caused by habitual physical inactivity (i.e., low metabolic-flux) driving decrements in the competitiveness of hepatic and skeletal muscle-cells in concert with the long-term failure of fat-cell plasticity and beta-cell function (DeFronzo, 1988; DeFronzo et al., 1992; Heilbronn et al., 2004; Galgani et al., 2008; DeFronzo and Tripathy, 2009).

## The Lack of Explanatory and Predictive Power of Gene- and Diet-Centric Paradigms

Science can be defined as the discovery of valid knowledge of the observable world. In contrast to other domains (e.g., philosophy and religion), science is distinguished by the capacity to explain, predict, and (where possible) control natural phenomena. Thus, the true test of scientific theories is how well they explain extant evidence. In this section we demonstrate that because the processes that lead to mammalian obesity and metabolic diseases are in fact well-established, anthropocentric speculations based on dietary and genetic correlations are inadequate [e.g., gene- and diet-centric models; see (Neel, 1962; Bray et al., 2004; Speakman, 2007; Swinburn et al., 2011)].

### Gene-Centric Paradigms Versus Non-genetic Evolutionary Processes

We posit that *accumulative maternal effects* are causal to variance in obesity and metabolic diseases independent of genotype (Archer, 2015c; Archer and McDonald, 2017). And because these non-genetic evolutionary processes mimic the alleged genetic effects, they provide a rigorous, mechanistic explanation for familial resemblance in metabolic and behavioral phenotypes (Archer, 2015a,b,c; Archer and McDonald, 2017; Archer et al., 2018). We previously commented on the lack of explanatory and predictive power of gene-centric paradigms (Archer et al., 2018), inclusive of epigenetics (Archer, 2015a,b,c). We stated that the “*missing heritability*…*will not be found in the genome*” (Archer, 2015c) but is explained almost entirely by *accumulative maternal effects* in the pre- and post-natal periods (Archer, 2015c; Archer and McDonald, 2017). These non-genetic evolutionary processes have significant and unequivocal effects on body and fat mass, and metabolic and behavioral phenotypes in offspring across species (Walton and Hammond, 1938; Falconer, 1965, 1967, 1973; Falconer et al., 1978; Liu et al., 1993; Maria et al., 1993; Brooks et al., 1995; Rossiter, 1996; Fox and Mousseau, 1998; Mousseau and Fox, 1998; Allen et al., 2002, 2004;Bonduriansky and Day, 2009; Mousseau et al., 2009; Freeman et al., 2013; Garg et al., 2013; Archer, 2015c; Archer and McDonald, 2017).

To extend our previous commentaries (Archer, 2015a,b,c; Archer and McDonald, 2017; Archer et al., 2018), we present several arguments. First, while the presence and initial location of mammalian fat-cells is clearly genetic (Raff, 2012), variation in the initial population of fat-cells (i.e., body cellularity) is determined almost exclusively by the amount of nutrient-energy reaching the placenta, intrauterine milieu, and fetus; which is controlled by the competition between maternal and fetal metabolic demands (Archer, 2015a,b,c,d; Archer and McDonald, 2017). A large body of observational and experimental research supports this argument. For example, monozygotic twins concordant for birth weight exhibit similar fat-cell numbers, while in those discordant for birth weight, the smaller twin displays both lower body mass and fat-cell number (Ginsberg-Fellner, 1981). Clearly, this effect is not genetic and is explained by chorionic status (Ramos-Arroyo et al., 1988; Cordero et al., 2005) and the intra-uterine competition for calories between the twins. The effect of intrauterine competition for energy substrates on fetal body and fat mass is extremely well-established in non-human animals, and inter-fetus competition due to litter size is the *“single greatest influence on birth weight”* (Gardner et al., 2007).

Second, as presented herein, obesity, and insulin resistance result from positive energy balance driven by fat-cell hyperplasia and/or physical inactivity (i.e., low metabolic-flux). Thus, we contend that the genes associated with obesity and metabolic disease are necessary but are not causal or predisposing factors. Association studies support our argument. For example, trends in physical activity over the past 50 years (Church et al., 2011; Archer et al., 2013b,c) explain cohort-specific associations of the FTO gene and obesity (Kilpelainen et al., 2011; Ahmad et al., 2013). Third, association studies are merely descriptive and provide no evidence of causality, whereas numerous experimental studies demonstrated the unequivocal and large consequences of *accumulative maternal effects* on metabolic outcomes (e.g., body and fat mass) and physical activity across species (Walton and Hammond, 1938; Falconer, 1965, 1967, 1973; Falconer et al., 1978; Liu et al., 1993; Maria et al., 1993; Brooks et al., 1995; Rossiter, 1996; Fox and Mousseau, 1998; Mousseau and Fox, 1998; Allen et al., 2002, 2004; Bonduriansky and Day, 2009; Mousseau et al., 2009; Freeman et al., 2013; Garg et al., 2013; Archer, 2015c; Archer and McDonald, 2017). For example, ovum transfer, animal breeding, and cross-fostering studies clearly demonstrated that the intrauterine milieu and early post-natal periods can induce obesity and metabolic dysfunction in a single generation, independent of genotype. Embryo transfer studies demonstrate that the inheritance of pathological metabolic phenotypes can be ameliorated or potentially abolished when the embryo is transferred and “*gestated in a normal metabolic environment”* (Garg et al., 2013). There are no studies that demonstrate similar genetic effects.

Fourth, as demonstrated over the past century, intrauterine exposure to reduced maternal metabolic control has significant effects on the health and metabolic trajectories of offspring (Freinkel and Goodner, 1962; Pedersen, 1967/1977; Whitelaw, 1977; Kervran et al., 1978; Catalano et al., 1998, 2003; Aerts and Van Assche, 2006; Egeland and Meltzer, 2010; Catalano and Hauguel-De Mouzon, 2011; Adamo et al., 2012; Cisse et al., 2013; Ferraro, 2013). For example, in siblings discordant for intrauterine exposure to T2DM, Dabelea et al. (2000, p. 22208) demonstrated that exposure *“conveys a high risk for the development of diabetes and obesity in offspring in excess of risk attributable to genetic factors alone”*. Similarly, Kral et al. (2006) showed that maternal weight-loss surgery reduced the prevalence of obesity and severe obesity in offspring by 52 and 45%, respectively. These studies show that maternal effects via altered maternal metabolism (i.e., altered metabolic-flux and inter-cellular competition) are causal to both obesity and T2DM. There are no similar studies demonstrating causal genetic effects. Finally, there is “a great deal of biology” between a nucleotide sequence and a phenotype, and there are myriad processes that render the associations between any given DNA sequence and a phenotype irrelevant [please see our prior work for a review (Archer et al., 2018)]. For example, alternative splicing and post-translational mechanisms can produce peptides with opposing physiological properties [e.g., the “Ghrelin Gene” (Zhang et al., 2005)].

In summary, these results support our contention that with respect to obesity and T2DM, genetic/epigenetic research is an incongruous level of analysis because “genes” are the “*tools of the* [species-specific cell]*, and their use (i.e., expression) is strictly environment-dependent*” (Archer, 2015b, p. 556). Thus, we posit that obesity and adult-onset metabolic diseases are exclusively environmentally induced phenotypes. These phenotypes have evolutionary consequences because in females, *accumulative maternal effects* induce the progressive inheritance of acquired characteristics, independent of changes to the genome.

### Diet-Centric Paradigms

Diet-centrism is the tendency to attribute a wide-range of negative health outcomes exclusively to dietary factors while neglecting the essential role of individual differences (Archer, 2018; Archer et al., 2018). The fundamental error of “diet-centrism” is the conflation of “diet” with nutritional status and health, in concert with the failure to acknowledge that identical diets consumed by different individuals result in divergent metabolic effects (Krogh-Madsen et al., 2014; Zeevi et al., 2015). The explicit conflation of diet with nutritional status and health contravenes the fact that the mammalian body is a complex ecosystem in which the effects of dietary factors are wholly dependent on the current state and compensatory fluxes of that ecosystem (e.g., metabolic phenotype and nutritional status). For clarity, an individual’s metabolic phenotype is influenced by factors, such as body cellularity and composition, nutritional status, physical activity and fitness levels, age, sex, reproductive, and disease status, and the state of the cellular systems responsible for metabolic control (i.e., skeletal muscle-, hepatic-, and pancreatic beta-cells) (DeFronzo, 1988; Archer, 2018).

Thus, it is not what is eaten (i.e., diet) that engenders health or disease, but what one’s body does with what was eaten (i.e., nutrient metabolism). Therefore, macro- and micronutrients cannot have health-effects independent of the metabolic phenotype of the consuming individual, and dietary components *per se* cannot be the determining factor in obesity and metabolic health (Archer, 2018). Thus, obesity and T2DM are not dietary concerns but are metabolic ones. Evidence in support of our argument is found across disciplines.

First, most diet-centric speculations are based on associations derived from data and methods previously demonstrated to be wholly invalid and scientifically *“inadmissible”* for the purposes of establishing causal relationships between dietary intake and health (Archer et al., 2015a,c, 2017b). Second, simple carbohydrates (e.g., dietary sugars and starches) and fats are often presumed to be causal factors, yet there are populations that consume substantial amounts of these macro-nutrients with very low prevalence of obesity and metabolic diseases (Ichikawa, 1981; Hill et al., 1984; WHO, 1995; Matsumura, 2001; Onywera et al., 2004; Marlowe et al., 2014). Therefore, “diet” is merely necessary, but not sufficient. Third, the prevalence of human obesity increased significantly across the globe in populations displaying dietary patterns differing in nutrient composition. Thus, dietary patterns are not causal. Fourth, there is no evidence that chronic positive energy balance is driven by the widespread availability of inexpensive, highly palatable foods and beverages. If this speculation was true, all humans in high income nations would be obese because these foods and beverages were ubiquitous for multiple generations. As such, fat-cell hyperplasia and/or physical inactivity (i.e., low metabolic-flux) induced increments in energy-intake behaviors provide a more rigorous, mechanistic explanation for over-nutrition.

Fifth, some of the strongest evidence to support our contention that dietary patterns and dietary components have no causal effect on the prevalence of obesity and metabolic diseases is inferential. Human dietary patterns cannot have caused the parallel increases in body and fat mass, obesity, and metabolic diseases in feral, laboratory, farm, and companion animals (i.e., rodents, horses, cats, and dogs) over the last half of the 20th century. Given the disparate environments and dietary patterns of these species, and the fact that all are placental mammals, *accumulative maternal effects* provides a more mechanistically rigorous explanation than diet-centric speculations based on mere associations.

Sixth, the strongest evidence supporting our contention that the diet-centric paradigm is mistaken is the well-established finding that over several generations, both obesity and metabolic diseases (e.g., T2DM, gestational diabetes) developed in non-human primates living in highly controlled environments with *“little to no change in diet, particularly in the rhesus and cynomolgus macaque species” (Bauer et al., 2010)*. The *“close genetic relatedness to humans*” (Bauer et al., 2011), make these species *“excellent models for* [obesity] *in humans” (Bauer et al., 2011)*. These results provide an unequivocal refutation of the diet-centric paradigm with respect to obesity and metabolic diseases (Archer et al., 2018).

Finally, diet-centric speculations cannot explain the rapid and differential increases in severe and morbid obesity (i.e., Class II and III) in adults and offspring during the late 20th century that vary by race, sex, and socio-environmental contexts (Sturm, 2007; Skelton et al., 2009). Conversely, the *Maternal Resources Hypothesis* and our frameworks provide a detailed, mechanistic, and parsimonious explanation for these population-specific trends.

### Unfounded and Unchallenged Conjecture Impede Progress

Scientific progress necessitates bold conjectures coupled with rigorous supporting evidence and comprehensive attempts at refutation. Nevertheless, within the domains of obesity and metabolic diseases, the sheer volume of unfounded and unchallenged conjecture threatens to obscure well-established evidence. Recently, we presented a review of the evidence that is contrary to the major etiologic paradigms and stated *“that progress in the understanding, prevention, and treatment of obesity and metabolic diseases requires moving beyond the epidemiologic ‘association-game’ in which mere correlations are cited as rigorous support for conjectures on causation”* (Archer et al., 2018). Nevertheless, there are hundreds of published speculations on the etiology of obesity and metabolic diseases ranging from air conditioning and vending machines, to viruses, mosquitos, and microbiota (Downey, 2015). These published putative “causes” rarely demonstrate any predictive or explanatory value, and none offer biologically plausible and non-trivial mechanisms in conjunction with rigorous experimental support. In fact, many speculations rely upon *“prescientific thought”* (Olesen and Alm, 2016) or tenuous correlations generated from *“pseudoscientific methods”* (Archer et al., 2015a,c, 2018), and are often demonstrative of *“physiologic illiteracy”* (Archer, 2018). Thus, we think the failure to distinguish between established causal mechanisms and mere speculations based on statistical associations continues to engender the proliferation of misleading and demonstrably false research programs and failed public health initiatives (Archer et al., 2017b, 2018; Archer, 2018).

In contrast, our novel frameworks in concert with our prior theoretical work represent a detailed, mechanistic, and comprehensive synthesis of rigorous experimental and observational results that spans the continuum from proximate to ultimate causation (i.e., physiologic and evolutionary, respectively). As such, this paper is a productive, albeit controversial step forward in constraining conjecture to hypotheses supported by well-established mechanisms.

## Future Directions

Our *Maternal Resources Hypothesis* and frameworks of a*symmetric nutrient-energy partitioning* and *effective caloric intake* are both retrodictive and predictive. Thus, unlike most conjectures, the ideas presented herein can be used to re-interpret and/or “predict” prior results while providing fodder for future investigations across myriad domains. Since all theories should be tested and have their foundational assumptions, background knowledge, and predictions challenged, we are currently planning a number of *in vivo* and *in silico* experiments to test our “body-as-an-ecosystem” and “cell-centric” approaches. Furthermore, given that our work spans multiple levels of analysis, we think empirical ventures targeting the evolutionary consequences of accumulative maternal effects on offspring cellularity and body mass over multiple generations are warranted. Additionally, we think examinations of the effects of body cellularity (e.g., ratio of high to low metabolically active cells) on cell-specific partitioning of lipids and glucose are potentially productive avenues for future research efforts.

## Conclusion

In this paper we presented the conceptual frameworks of *asymmetric nutrient-energy partitioning* and *effective caloric intake.* These frameworks, in concert with our previous theoretic work, the *Maternal Resources Hypothesis*, provide a parsimonious and physiologically rigorous explanation for the rapid rise of the global prevalence of obesity and metabolic diseases in human and other mammalian species.

## Author Contributions

Each author contributed to the intellectual content and organization of this paper. EA wrote the paper with assistance from each of the co-authors.

## Conflict of Interest Statement

Dr. EA is employed by Evolving*FX*, a data analytics company. The remaining authors declare that the research was conducted in the absence of any commercial or financial relationships that could be construed as a potential conflict of interest.
